# The roads towards fidelity: a mixed-method study of mechanisms of a multifaceted implementation strategy to improve implementation of a guideline for the prevention of mental Ill-health at the workplace in a school setting

**DOI:** 10.1186/s43058-026-01010-0

**Published:** 2026-06-11

**Authors:** Andreas Rödlund, Rebecca Lengnick-Hall, Anna Toropova, Byron J. Powell, Liselotte Schäfer Elinder, Christina Björklund, Lydia Kwak

**Affiliations:** 1 Unit of Intervention and Implementation Research for Worker Health, Institute for Environmental Medicine, Karolinska Institutet, Stockholm, 171 77 Sweden; 2 Center for Mental Health Services Research, Brown School of Social Work, Washington University in St. Louis, St. Louis, MO USA; 3 Dissemination and Implementation Science Innovation Research Network, Bursky School of Public Health, Washington University in St. Louis, St. Louis, MO USA; 4 Division of Infectious Diseases, John T. Milliken Department of Medicine, School of Medicine, Washington University in St. Louis, St. Louis, MO USA; 5 School of Public Health, College of Medicine & Health, University College Cork, Cork, Ireland; 6 Department of Global Public Health, Karolinska Institutet, Stockholm, 171 77 Sweden; 7 Centre for Epidemiology and Community Medicine, Region Stockholm, Stockholm, 104 31 Sweden

**Keywords:** Implementation strategies, Mechanisms of change, Evidence-based practice, Mental health problems, Psychosocial risk management, Theory-based evaluation methods, Process tracing, Qualitative comparison analysis

## Abstract

**Background:**

Many implementation studies focus on assessing the effectiveness of implementation strategies without investigating the mechanisms explaining their effects. This study investigates the specific mechanisms through which a multifaceted implementation strategy is hypothesized to improve guideline fidelity. This is done by tracing the specific mechanisms through which each discrete strategy achieved proximal outcomes that precede guideline fidelity.

**Methods:**

Process tracing with comparative case studies was used to analyze qualitative data from a subsample of 16 implementers participating in a cluster-randomized controlled trial exploring the mechanisms by which a multifaceted strategy affects fidelity to a guideline for prevention of mental problems in schools. The study involved four steps: (1) Organizing a traceable process theory of change; (2) Gathering data to validate, modify, or expand the theory; (3) Coding data through qualitative content analysis; and (4) Causal analysis to articulate the theory’s mechanisms, including temporal order and key contextual conditions that explain success or failure. The unit of analysis was implementers who were members of the schools’ implementation teams, with each implementer representing a case.

**Results:**

The analyses illuminated distinct pathways that explain how each of the five discrete strategies led to implementation success. (1) Organizing Implementation Teams enabled participation in implementation activities, as principals created an environment with dedicated resources, enabling social influences; (2) Educational meetings influenced the decision to implement the guideline through the educators’ who promoted knowledge acquisition among the implementers and shaped their beliefs about the implementation; (3) Ongoing Training influenced the intention for implementation and enactment of activities through educators’ engaged processes around ongoing acquisition and refinement of skills, which gradually built belief in implementation capability; (4) Small Cyclical Tests of Change influenced behavioral regulation as the workshops engaged repeated engagement in goal setting, action planning, reflection, and adjustment; (5) Facilitation supported implementers’ goal setting and prioritization by creating an environmental context, enabling implementers’ social influence through receiving social support from facilitators. Unsuccessful pathways for each strategy were also identified and explained.

**Conclusions:**

This study traced the specific sequences of actions and interactions through which five discrete implementation strategies contributed to guideline implementation. The findings illustrate how the mechanisms of the multifaceted strategy can be successfully engaged and why they may fail under certain conditions.

**Trial registration:**

The trial was registered the 9th of August 2021 at Clinicaltrials.gov with Trial registration number: NCT05019937

**Supplementary information:**

The online version contains supplementary material available at 10.1186/s43058-026-01010-0.

Contributions to the literature

- Findings illustrate how five commonly used discrete implementation strategies function when embedded in a multifaceted strategy.
- This study identified mechanisms by combining in-depth within-case analysis and cross-case comparison. By tracing potential sequences of actions and examining recurring patterns, deviations, and contextual conditions, we clarified which processes were necessary for the strategies to work and under what circumstances.
- This study presents a rigorous methodological approach that illustrates how qualitative methods can be used to study mechanisms of implementation strategies.

## Background

Implementation strategies aim to promote the implementation of evidence-based practices (EBPs) across various domains, including education and occupational health and safety [1-3]. There is consensus that multifaceted implementation strategies are needed in complex, multi-level settings such as schools, addressing multilevel determinants in a structured sequence [4, 5]. For example, one strategy may target a determinant at one level (e.g., school-level) to increase the responsiveness of another discrete strategy operating at a different level (e.g., implementer-level) [6, 7]. While research on multifaceted strategies has primarily focused on their relative effectiveness compared to discrete strategies [8–10], fewer studies have examined the mechanisms by which these strategies address determinants and exert their effects [11, 12].

An implementation strategy mechanism is defined as “*the process responsible for change*” and explains how and why strategies affect intended outcomes [13]. According to Glaser and Laudel, mechanisms can be conceptualized as sequences of causally linked actions and interactions through which change unfolds [14]. Accordingly, implementation strategies operate by engaging specific mechanisms that drive change toward an implementation outcome. Understanding mechanisms can inform the design of more effective strategies, guide the bundling and sequencing of strategies, and enhance the impact of existing multifaceted strategies, ensuring that each component has the greatest potential to influence intended determinants and outcomes [15–17].

An increasing number of studies have statistically tested how implementation strategies achieve outcomes, including tests of mediators articulated within theories of change [18, 19]. While such studies contribute to the understanding of mechanisms by identifying mediators that both statistically and theoretically account for the relationship between specific strategies and outcomes, they provide less granularity about how change unfolds, who changes and when, who does not change, and under what conditions [20]. This is partly due to reliance on experimental and statistical models that assume linear change [21]. However, implementation processes are usually non-linear, iterative, and shaped by contextual conditions [20–22]. To address this, there have been calls for alternative methods to study implementation strategy mechanisms, including qualitative and case-based methods [13, 15, 23]. This study responds to this call by applying a qualitative, case-based approach to examine implementation strategy mechanisms, focusing specifically on how a multifaceted implementation strategy operates, and tracing the sequences of causally linked actions and interactions through which change unfolds.

### The current study

This study is an empirical investigation embedded within a cluster-randomized controlled trial to explore the mechanisms through which a multifaceted strategy operates to affect fidelity to the *Guideline for the Prevention of Mental Ill-health at the Workplace* in Swedish schools [24]. The multifaceted strategy includes five discrete strategies: organizing an implementation team, an educational meeting, ongoing training, small cyclical tests of change, and implementation facilitation [24]. Each discrete strategy represents a causal input (or cause) that was intentionally selected to address pre-identified determinants and engage specific mechanisms, and, in combination, contribute to guideline fidelity [24, 25]. Findings from two cluster-randomized trials conducted in Swedish schools showed that this multifaceted strategy improved guideline fidelity over 12 months [26, 27]. In the most recent trial, in which this study is embedded, statistically significant differences were observed compared to a discrete strategy [27].

**Fig. 1:**
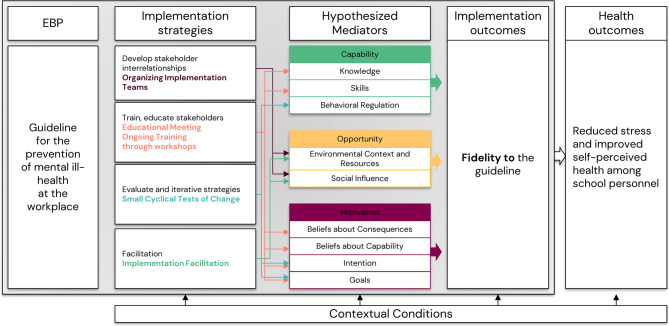
The program theory for the multifaceted strategy. Note: the program theory is reproduced and slightly modified from the study protocol [24] for this trial

The original program theory explaining how the multifaceted strategy leads to improved guideline fidelity is illustrated in Fig. 1. This program theory draws on the COM-B model [28] and the Theoretical Domains Framework (TDF) [29]. It proposes that the multifaceted strategy equips implementers of the guideline with the capability, opportunity, and motivation to implement the guideline [28]. The TDF specifies domains that influence implementation [29], with eleven domains focusing on the individual-level and three domains reflecting contextual influences [30]. As shown in Fig. 1, nine TDF domains were identified as hypothesized mediators of the multifaceted strategy’s effect on fidelity and were mapped onto capability, opportunity, and motivation to provide the pathways for change. When the effectiveness of the multifaceted strategy was tested in the trial, eight out of nine of the TDF domains partially mediated the effect of the multifaceted strategy on the primary outcome guideline fidelity at 12-months [19]. The effectiveness of the multifaceted strategy, however, depends on the combined effect of its discrete strategies on more proximal outcomes that precede guideline fidelity. These proximal outcomes resemble the TDF domains that the discrete strategies are intended to address as depicted in the trial’s program theory (Fig. 1). Even though the findings of our previous trial provided valuable evidence for multiple mechanisms of the multifaceted implementation strategy, it was not possible to disentangle which discrete strategy engaged which mechanism, in what sequence, and under which contextual condition. To address this, the present study will specify, through Process Theories of Change (PTOC), how each discrete implementation strategy is intended to engage specific mechanisms. The PTOCs for each discrete strategy will accordingly be tested using process tracing. This will enable us to empirically trace how these mechanisms unfold through sequences of actions and interactions to intended proximal outcomes under specific contextual conditions [31, 32]. Moreover, it will increase our understanding of how the strategies combined contribute to guideline fidelity. While our prior work focused on testing mediators (operationalized as single TDF domains), the mechanistic focus of this paper positions mediators within a broader, more holistic causal pathway that unfolds over time.

### Study aim

This study aimed to explain how and why the multifaceted strategy worked by identifying the mechanisms engaged by its five discrete strategies. Three objectives guide this study:

1. To identify the mechanisms and their constituent sequences through which each discrete implementation strategy operates.
2. To determine which of these sequences are necessary to achieve the discrete strategies’ intended outcomes.
3. To investigate the contextual conditions that influence how these mechanisms and sequences unfold.

## Methods

### Study context

The cluster-randomized controlled trial in which the study is embedded compared the multifaceted strategy with a discrete strategy [24]. The guideline to be implemented promotes a systematic approach to the identification of workplace psychosocial risks, followed by the development, implementation, evaluation, and follow-up of action plans to reduce these risks [33]. Between 2021 and 2023, the guideline was implemented in 55 schools across four municipalities. Twenty-nine schools received the multifaceted strategy between 2021 and 2022 (ARM 1), and 26 schools between 2022 and 2023 (ARM 2). At each school, local implementation teams were formed, containing between 3 - 5 members. Teams participated in an educational meeting, and five workshops, which were led by the research team. The implementation teams were responsible for implementing the guideline within their school by using small cyclical tests of change, operationalized through the plan–do–study–act (PDSA) methodology [34]. To support the implementation teams, each municipality appointed internal facilitators at the municipal level. This study focuses on the implementers who received the multifaceted strategy between 2021 and 2022. Prior work from this trial described the effects of the multifaceted strategy on guideline fidelity through identified mediators, operationalized as TDF-domains [19]. This study extends that work by examining how the discrete strategies influence the TDF domains they are hypothesized to address (see Fig. 1 for hypothesized links between strategy and mediator). Moreover, the mechanistic focus of this paper positions these TDF domains within a PTOC for each strategy that specifies the temporal ordering through which they unfold within a causal pathway. Specifically, this study specifies these domains through the sequences of actions and interactions through which the discrete strategies operated, and by examining the contextual conditions that shaped how these sequences unfolded. It also enables the identification of previously unidentified mechanisms and contextual conditions that more accurately explain how the strategy works.

### Overall study approach

This study applied process tracing with comparative case studies [14] (see Table 1). In Step 1, the program theory (Fig. 1) was reorganized into a PTOC to make it suitable for process tracing [32, 46]. A PTOC disaggregates a mechanism into sequences, where each sequence consists of actions and interactions that together explain how change unfolds. The mechanism is engaged by the implementation strategy and leads to a specific proximal outcome [40]. This step provided the starting point for gathering and analyzing data [24, 25]. In Step 2, data were gathered to validate, modify, or expand the PTOC. Three sources of data were used for each case: semi-structured interviews, PDSA documents, and attendance lists from educational meetings and workshops. In Step 3, Extractive Qualitative Content Analysis (EQCA) [42] was applied to organize and structure the data by coding and sorting it into categories defined by the PTOC, where the sequences and outcomes of each discrete strategy represented a separate category. This version of qualitative content analysis was used because it organized information on the causes and effects of the categories, enabling exploration of the interplay between categories in Step 4 [14]. In Step 4, causal reconstructions were made within each case to create case overviews from the organized material. These overviews were used to compare different cases and identify recurring (or typical) patterns, deviations, and influential contextual conditions. This comparison formed the interpretative basis for Qualitative Comparative Analysis (QCA), which determined the sequences and contextual conditions necessary and sufficient for strategy success or failure [43, 47]. The unit of analysis was implementers, which aligned with the trial’s theoretical foundation of COM-B and TDF. Table 2 presents an overview of the terminology used in this study.

**Table 1:** The justification and value added of the four study steps

	Justification (why this step was necessary)	Value added (what this step provides)
**Overall approach**	Process tracing provides a detailed within-case method for examining how a causal mechanism operates in a case [35, 36]. Relying only on single cases, however, risks producing insights that may be too narrow. By supplementing this with comparative case studies, we can assess whether the same mechanism is visible in other cases and what contextual conditions its operation depends on [14].	This overall design gives us both depth and breadth: we learn how the strategy produces its effects over a population of cases, and we also learn why the effect may vary depending on the presence or absence of contextual conditions. This makes our causal claims stronger and more generalizable [14, 35, 36].
**Step 1:** The Process Theory Of Change (PTOC)	Theory-based evaluation starts from the idea that a strategy should be tested against how it is expected to work [37–39]. We organized our program theory into a traceable PTOC. This was necessary because the function of the discrete strategies was hidden in assumptions [32]. This theory provided a language that makes these assumptions explicit and traceable by unpacking the actions of actors involved in the strategy [32].	This provided the starting point of the study, which gave us a road map that can be tested empirically, thereby validating, modifying, or expanding it through empirical evidence. An empirically supported theory increases the credibility of the causal claims and generates transferable knowledge once the contextual conditions for its operation are also known [32].
**Step 2:** Gathering Data	Process tracing requires empirical material to test whether the theorized mechanism occurred as expected, unfolded differently, or failed to appear [35, 36]. This step involved gathering evidence, including documents and interviews, to assess whether the causal process hypothesized in the theory was evident in a case and the population cases.	Using multiple sources strengthens the credibility of the findings by allowing corroboration and complementarity [40]. This increases confidence that the evidence reflects the theory of change, rather than the biases or limitations of any single source [35, 40].
**Step 3:** Organizing Data	Once the data were collected, they needed to be organized to allow for structured and focused analysis of cases [41]. We used Extended Qualitative Content Analysis (EQCA), which organizes information by coding and subsuming text into categories derived from the theory of change [14, 42]. This makes the material comparable across cases. To identify mechanisms, causal information is also needed. EQCA supports this by organizing the causal information within each category (input and output to the category).	EQCA produces a reduced and structured information base tailored to answer the research questions [14, 42]. This enabled a structured and focused analysis, where evidence from each case is sorted in a comparable way through the same conceptual lens. The organization of causal information further facilitates the reconstruction of processes, as it facilitates the recombination of information to rebuild the causal processes [14, 42].
**Step 4:** Causal Analysis	The causal analysis answered the research questions [14]. First, we conducted a causal reconstruction within each case to create case overviews. This involved assembling and combining categories from the EQCA to carve out the causal process in each case [14]. Second, we compared typical and deviant cases to identify recurring patterns, deviations, and contextual conditions. This enabled us to articulate the candidate mechanism for each strategy and specify the contextual conditions that shaped its operation [14]. Finally, we used QCA to analyze all cases together and determine the conditions and combinations of conditions that were linked to the outcome and failed outcomes [43].	This step enabled us to understand how each strategy unfolded for individual implementers and how these processes varied across cases. By adding cross-case comparison, we could carve out a common timeline for the population of cases and their contextual conditions [14]. QCA is further useful in combination with case study analysis [43, 44]. Case studies provide familiarity with cases and ensure interpretation of QCA results, while QCA offers precision in determining relevant similarities and differences by clustering cases into different paths toward the outcome and failed outcome [45].

**Table 2:** Terminology

Terminology	Descriptions	Study conceptualization
Theory-based evaluation	Theory-based evaluation typically begins with a program theory and use empirical data to investigate how mechanisms unfold in practice, enabling refinement of the theory [37, 38].	In this study, the program theory of the multifaceted strategy is tested and refined through process tracing.
Program theory	Program theory refers to a set of explicit or implicit assumptions about what action is required to solve a social, educational, or health problem, and why the problem is expected to respond to this action [37].	In this study, the program theory specifies how the multifaceted strategy is expected to improve guideline fidelity in schools (see Fig. 1).
Process theory of change (PTOC)	A PTOC is a form of theory of change that builds on a system understanding of a mechanism that disaggregates the mechanism into two or more sequences [32].	In this study, the PTOC specifies how each discrete strategy unfolds through sequences of actions and interactions to reach a traced outcome (see Fig. 2).
Cause	The causal input (X) that engages a mechanism [32].	In this study, the implementation strategy (e.g., educational meeting) is conceptualized as the cause that engages a mechanism.
Mechanism	A mechanism refers to “the process responsible for change” [13] through which an implementation strategy leads to an outcome.	In this study, a mechanism is conceptualized as the underlying causal process triggered by an implementation strategy, through which change occurs. Mechanisms unfold through sequences of actions and interactions that together produce the traced outcome.
Sequence	A sequence refers to an analytically defined phase within a mechanism, consisting of causally linked actions and/or interactions that together move the process forward [35, 36].	In this study, a sequence represents a distinct phase within a mechanism, consisting of linked actions and interactions that move the causal process forward.
*» Action*	A specific activity performed by an actor that contributes to the progression of a sequence, such as providing education or setting goals [32, 35].	In this study, an example is educators delivering a lecture on the guideline.
*» Interaction*	A relational process in which one actor’s action influences another actor’s response, thereby contributing to the progression of a sequence [32, 35].	In this study, an example is that implementers reflect on information provided by educators and adjust their plans.
*» Actor*	Those involved in the causal process [32, 35].	In this study, actors include principals, implementers, educators, and facilitators
Outcome	The output (Y) of the theorized pathway that is traced [35, 36].	In this study, outcomes refer to proximal outcomes that occur before the primary implementation outcome fidelity.
Implementation outcome	Implementation outcomes (e.g., fidelity) refer to the effects of deliberate actions to implement an evidence-based practice [48].	In this study, fidelity is the primary implementation outcome of the multifaceted strategy, while the present analysis focuses on proximal outcomes that precede fidelity.
Contextual Condition	A contextual condition is a characteristic or circumstance that shapes how a mechanism operates and influences whether and how sequences unfold, thereby affecting the likelihood of achieving the outcome [35, 36].	In this study, contextual conditions are factors that shape whether and how mechanisms unfold. They influence the activation and progression of sequences. For example, the presence of cross-level collaboration.
Pathway	A timeline of how the process unfolds, including the cause, mechanism, and outcome, accompanied by the contextual conditions that need to be present [35, 36].	In this study, a pathway represents the empirically observed progression from strategy (cause) through the sequences of a mechanism to an outcome, including the contextual conditions that shape this process.
Categories	Generalized descriptors that allow delineation and grouping of the empirical data [14].	In this study, categories include constructs such as knowledge, beliefs about consequences, and social influence (see Additional file 1).
Causal reconstruction	Causal reconstruction means looking back to map the sequence of action/interactions that led to a particular outcome [41].	In this study, this involves reconstructing how implementers moved from strategy exposure to outcome through specific sequences.
Sufficient condition	A sufficient condition is a condition that, when present, is consistently associated with the outcome. This means that whenever the condition is present, the outcome is also present, although the outcome may also be achieved through other conditions or combinations of conditions [36].	In this study, sufficient conditions may include specific sequences or contextual conditions that, when present, lead to an outcome. Although the same outcome may also occur through other sequences or contextual conditions. For example, knowledge acquisition may lead to maintained intention for implementation, although maintained intention may also occur without knowledge acquisition.
Necessary condition	A necessary condition is a condition that must be present for the outcome to be achieved. In its absence, the outcome cannot be achieved [36].	In this study, necessary conditions may include specific sequences or contextual conditions required for outcome attainment. The outcome will not occur without these conditions. For example, implementers needed to engage in goal setting and action planning for the proximal outcome of behavioral regulation to occur.

### Step 1: Process theory of change

**Fig. 2:**
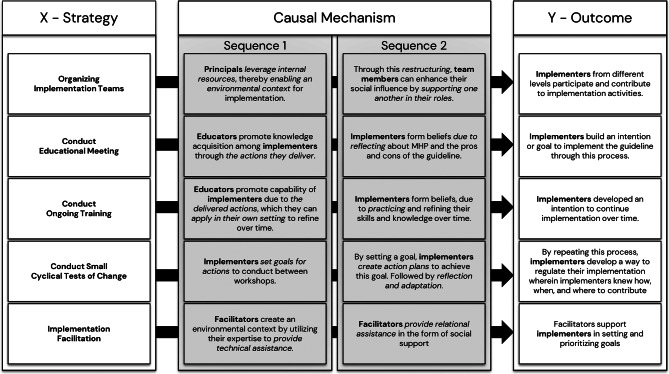
Shows the initial PTOC. Actors in the pathways are marked in bold text, and actions are presented in *italicized* (*curved*) text

First, each discrete strategy was defined as a cause (i.e., the causal input that engages the mechanism [32]), thereby providing the starting point for tracing five pathways within the multifaceted strategy. Second, the outcomes and mechanisms were defined for each strategy. The study protocol [24] was revisited to define outcomes and mechanisms, with each strategy described in terms of actors, actions, and targeted TDF domains. This information was reorganized into a temporal order. To ensure consistency with our prior work [19, 24], the most distal TDF domain targeted by each strategy was defined as the strategy’s proximal outcome. While some of these outcomes may overlap conceptually with implementation outcomes as defined by Proctor and colleagues (e.g., adoption or appropriateness) [48], this conceptualization ensured alignment with the original program theory of our multifaceted implementation strategy (Fig. 1). Thus, each strategy was linked to a unique outcome to be traced (see Fig. 2) [19]. The mechanisms were defined by drawing on the COM-B model to propose a potential temporal ordering of the pathways, after which TDF constructs were mapped onto capability, opportunity, and motivation. Additional literature, including methodological papers (e.g. [49–53]), systematic reviews (e.g. [50–53]), and meta-analyses (e.g. [54–56]), was further used to support these pathways. Based on this information, pathways were disaggregated into actions and interactions by actors. Finally, contextual conditions were theorized through COM-B’s component ‘Opportunity’. Implementers’ opportunity was expected to shape how mechanisms unfold and was operationalized through its related TDF domain of environmental context and resources and social influence. An example of how the program theory was translated into a PTOC, including the sequencing of mechanisms and their disaggregation into actions and interactions, is presented in Table 3.

**Table 3:** Example of how the program theory was translated into a PTOC

Study Protocol (Educational Meeting) – Program theory			
Action(s)	Actor(s)	COM-B	TDF
**Educate on**: → Mental ill health → The guideline → The advantages of adhering to the guideline. **Facilitate**: → Goal formulation through exercises.	Research team, Implementers	Capability, Motivation	Knowledge, Beliefs about Consequences, Beliefs about Capability, Intention, and Goals
**Process Theory of Change (Educational Meeting)**			
**Cause** →	**Sequence 1** →	**Sequence 2** →	**Outcome**
**Step 1** (Sequencing)			
Educational Meeting	Capability	Motivation	Behavior (Outcome)
**Justification:** Capability influences motivation to enact behavior. Motivation influences behavior [28].			
**Step 2** (Mapping domains)			
Educational Meeting	Knowledge	Beliefs about Consequences and Capability	Intention, Goals
**Justification:** Provision of education and information on how to perform behavior influences knowledge [49, 52, 53]. Knowledge about health consequences and pros and cons influences beliefs (e.g., attitudes and perceived behavioral control) [53]. Perceived behavioral control and attitudes influence intention [51, 54, 57].			
**Step 3 (**Disaggregated to actors’ actions and interactions)			
Educational Meeting	**Educators** promote knowledge acquisition among **implementers** through *the actions they deliver*	**Implementers** form beliefs *due to reflecting* about MHP and the pros and cons of the guideline.	**Implementers** build an intention or goal to implement the guideline through this process.
**Step 4** (contextual conditions)			
Contextual conditions	Opportunity Environmental Context and Resources	Opportunity Social Influence	

### Step 2: Gathering data

Data were collected between September 2021 and March 2023 via semi-structured interviews, documents, and attendance lists. The primary data source was semi-structured interviews. Documents and attendance lists were used to supplement and contextualize statements from the interviews.

For the semi-structured interviews, a purposive sampling strategy was initially used to recruit implementers with experience of the implementation process who could provide information about the mechanisms of specific strategies. Potential participants were identified by the research team to capture variation in experience of the implementation process, including both those for whom it worked well and those who encountered difficulties, to gain an understanding of important contextual conditions [58, 59]. The identified implementers (*n* = 12) were invited to participate via email; six accepted, one declined (due to lack of time), and five did not respond to the initial invitation or two reminders. Ten additional implementers were recruited through a snowball sampling strategy, in which the implementers interviewed recommended the next individual to interview.

Interviews were conducted between November 2022 and March 2023 using Microsoft Teams [60]. The implementers represented a diverse range of occupational roles, including principals, school administrators, and teachers acting as health and safety officers, union representatives, and team leaders. The interviews were recorded digitally and transcribed verbatim by a professional company. One researcher (AR) conducted the interviews using a semi-structured interview guide. LK, CB, or AT participated in the initial ten interviews to observe and provide feedback on the interview technique, without intervening in the interview situation. Inclusion criteria for this study were implementers who had received the multifaceted strategy and had participated in at least half of the workshop series to ensure participants were exposed to the core content of the strategy. The unit of analysis in this study was the individual implementer, with each implementer treated as a separate case. The 16 implementers came from 11 different schools (implementation teams). Eight implementers were from three schools (with three implementers from one school, three from another, and two from a third), while the other eight were the only representatives from their school.

The documents collected were the PDSA documents, developed by the implementation teams during the implementation period. On average, each team developed four documents. All documents [61] were structured in the same way for the teams to describe their implementation goal using the SMART format (Specific, Measurable, Achievable, Realistic, and Timely), their implementation plan (who, what, when, and where), and their progress across the steps of the PDSA cycle. Additionally, the research team collected attendance lists from the educational meeting and the five workshops to provide a concrete record of participation.

### Step 3: Organizing data

EQCA [14] was then used to organize this case material. This involved developing categories (see Table 2) based on the PTOC, coding interview text into these categories, and consolidating the text [14]. First, elements of the PTOC (Step 1) were translated into categories, defined as generalized descriptors that allowed for delineation and grouping of the empirical information [14]. Each category was multidimensional to capture its content, as well as what caused it (input) and its effect (output) (Appendix 1). Categories were further named in accordance with the TDF constructs [29] to ensure a connection to previous work [19].

AR then coded text from the interviews into the above constructed categories. When relevant information did not fit the predefined categories, it was extracted and saved separately and coded into categories in the next round [42]. The initial coding was checked in peer debriefing meetings with AR, AT, and LK by selecting excerpts (extracted text), which were then sorted into categories (blinded to the initial categorization) to ensure agreement about category coding. The interview data were then supplemented with document review. Documents were used to clarify (e.g., interviews described two sequences and documents clarified their temporal order) and contextualize interview accounts [40, 46].

Following initial coding, three main adjustments were made in the categories. First, the categories of environmental context and resources, and social influence, which were intended to capture contextual conditions, were considered too broadly defined and were divided into subdimensions (see Appendix 1). Two new categories were added based on unexpected information: a category of social/professional role and identity [29] was added, as the data also reflected a motivation to participate among implementers rather than just an opportunity to participate. In discussions between the co-authors, a decision was also made to add enactment as an additional category to reflect that implementation had been carried out. After these adjustments in categorization, the data were re-assessed using the revised categories. The second round of coding was reviewed and discussed. Following the second round, the material was summarized to capture the key actions, actors, and inputs and outputs within each category. The material was then organized by case and strategy to facilitate step 4.

### Step 4: Causal analysis

#### Creating case overviews to identify mechanisms

Drawing on the coded material from Step 3, information within each case was reconstructed to create case overviews for each strategy. This involved combining information across categories to reconstruct the sequences within each case. For example, the causal input of one category was linked to the content of another, and the effect of that category was then connected to the subsequent content of another category. This information was then condensed into case overviews with timelines for each case and strategy, supplemented with short written descriptions that highlighted how each sequence unfolded.

#### Using case overviews to derive mechanisms

Following Glaser and Laudel’s [14] procedure, the case overviews were compared and organized into a cross-tabulation of cases against the mechanism’s sequences, allowing sequences across cases to be examined side by side. The comparison began with the cases that achieved the outcome, which revealed recurring sequences that all typical implementers went through to reach the strategy outcome, representing a candidate mechanism. Several of the initially theorized sequences did not unfold as expected or were deviant when compared to the typical cases. For example, ongoing training was hypothesized to engage both knowledge and skill acquisition. While knowledge acquisition during the workshops was observed in some cases, it was not observed across all typical cases. Similarly, in the small cyclical tests of change strategy, implementers described two types of pathways: one that adhered to the PDSA structure and another that omitted the “Study” and “Act” phases of the PDSA cycle. Next, deviant cases were analyzed [14]. Deviant cases were also checked for contextual conditions that hindered the mechanisms. This provided a common timeline for how each strategy successfully unfolded (or failed to), which was the interpretive foundation for the QCA.

#### Confirming the mechanisms

The final step confirmed the pathways by analyzing all cases together [14]. Qualitative Comparative Analysis (QCA) was applied to determine whether the identified sequences and contextual conditions were consistently necessary across cases, thereby enabling us to distinguish them from sufficient ones. A necessary condition is a sequence or contextual condition that must be present for the outcome to occur, without the condition the outcome will not occur. A sufficient condition is a sequence or contextual condition that is consistently present when the outcome occurs, however the outcome may also occur without the condition being present [43, 44]. Using crisp-set QCA, sequences and contextual conditions were coded as present or absent in a raw data table based on the case overviews [47]. The raw data table was transformed into a truth table, and its quality was assessed based on variation across cases, conditions, and outcomes; contradictory configurations of conditions; and the reliability of the qualitative coding for presence or absence of conditions [45]. For each strategy, individual conditions and combinations of conditions needed for outcome attainment were first analyzed. To understand what led to the mechanism not being engaged, an examination was also conducted on what conditions were present in cases that failed to achieve outcomes. This produced a solution of necessary conditions for outcome attainment and failure [45]. QCA analyses were conducted in TOSMANA version 1.61 [62].

## Results

In the following sections, we describe the successful and unsuccessful pathways for each of the five implementation strategies. Table 4 presents the sample characteristics.

**Table 4:** Study sample with demographics and their role in their school

Demographics	N
**Job Role**	
Principal	9
School administrator	2
Health and Safety Officer	1
Union Representative	3
Team leader	1
**Gender**	
Female	10
Male	6

**Note:** The roles of health and safety officer and union representative are positions of trust, and the individuals holding these roles are also teachers by occupation

### Strategy 1: Organizing implementation teams

The outcome of this discrete implementation strategy was that implementers from different organizational levels participated and contributed to implementation activities. Implementers’ accounts showed that this reflected a shared responsibility for activities across school management and staff: “*I do not feel like I was the one leading it forward, but rather that we were all leading … Everyone contributed equally, so to speak*” (IP 12). These accounts were echoed in the attendance lists, which confirmed that staff with different occupational roles and from organizational levels participated. For this strategy, three distinct pathways were identified: one successful (*n* = 12) and two unsuccessful pathways (*n* = 2 and *n* = 2).

#### Successful pathway

**Fig. 3:**
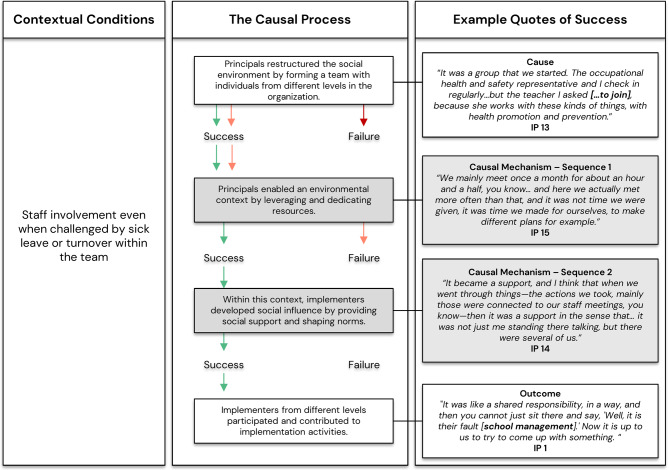
The pathways for organizing implementation teams. Note: the successful pathway is illustrated with green arrows, while the two unsuccessful pathways are illustrated with orange and red arrows, respectively. Success = presence; failure = absence. Each part of the pathway is accompanied by an example quote from the data

Figure 3 depicts the pathways of the ‘organizing implementation teams’ strategy.

One contextual condition was necessary (i.e., must be present for the outcome to occur) for the strategy to function: staff involvement (see Fig. 3). The strategy engaged the mechanism when principals restructured the social environment by forming a team comprising individuals from different organizational levels. This restructuring involved selecting members from management and staff to bring together diverse occupational roles and levels, thereby establishing a team structure for shared responsibility. Following this, the mechanism unfolded through two sequences. First, through this restructuring, principals enabled a dedicated context for discussing, planning, and implementing activities by leveraging internal resources (e.g., time from regular work assignments). One implementer described: “*When we formed the team, it led to more meetings together and more focus specifically on this work, which in turn resulted in a more systematic way of working*” (IP 4). Document review confirmed that teams planned meetings and allocated time for the implementation. Second, within this context, implementers provided social support to each other, shaping norms around shared responsibility for implementation: *“… he [the principal] got help, you know, he might have been alone in this work. But now he’s not alone anymore; now he’s the one with the overall responsibility, but he gets input, you know … so, in that way, I think it has contributed*” (IP 2).

#### Unsuccessful pathways

Two unsuccessful pathways were identified, explaining why some implementers failed to complete the ‘organizing implementation team’ strategy. In the first, principals restructured the social environment by forming a team but failed to establish a dedicated context: “*Unfortunately, it … did not lead to us remembering or prioritizing working with the document in everyday practice … the group did not prioritize or meet regularly at school*” (IP 8). In the second unsuccessful pathway, principals were unable to form a team with individuals from different levels in the organization: “*It has been a bit up and down … I have also been quite alone in this*” (IP 5). In these cases, sick leave and turnover were present, and without a team that included individuals from different organizational levels, the outcome was not achieved.

### Strategy 2: Educational meeting

The traced outcome of this strategy was the decision to implement the guideline. Implementers’ accounts showed how the meeting enacted the development of an initial plan or goal to implement the guideline: “*It ended up more on the agenda because we addressed it right away … as I recall, we concluded that we needed to have some kind of plan*” (IP 13). For this strategy, two pathways were identified: one successful (*n* = 13) and one unsuccessful pathway (*n* = 3).

#### Successful pathway

**Fig. 4:**
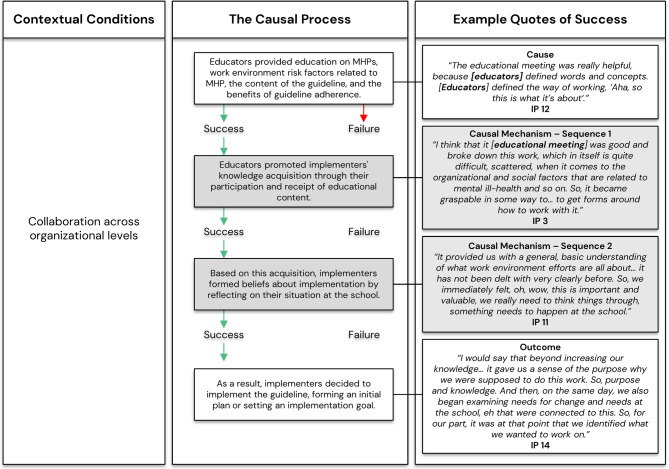
The pathways for the educational meeting. Note: the successful pathway is illustrated with green arrows, while the unsuccessful pathway is illustrated with red arrows. Success = presence; failure = absence. Each part of the pathway is accompanied by an example quote from the data

Figure 4 illustrates the pathway that the implementers went through to decide to implement the guideline.

In this pathway, the contextual condition of social influence was present, including collaboration across organizational levels, which supported the decision to implement the guideline. The strategy engaged the mechanism when educators provided education on work-related MHP and associated risks, the guideline content, and the benefits of guideline adherence. Following this, the mechanism unfolded through two sequences. First, the educators promoted implementers’ knowledge acquisition, which enabled an awareness and understanding of the guideline and what guideline implementation involves, which implementers could use when deciding to implement the guideline: “*The educational meeting helped us get ourselves on track in relation to the guideline and how we could work with it during … , well, the coming year*” (IP 15). Second, implementers engaged in reflection on their own situations based on this knowledge, which shaped their expectations about implementation. This led implementers to recognize the relevance of the guideline and the necessity of implementation: “*It was when we had received our results about the work environment, which was orange [reflecting risks]. And then we felt, well, this [Guideline implementation] could be a help along the way*” (IP 6). This sequence reflected that implementers held positive outcome expectancies regarding the work environment management through the implementation of the guideline.

#### Unsuccessful pathways

In the unsuccessful pathway, the strategy failed to engage the mechanism. Specifically, the provision of education did not lead to knowledge acquisition among implementers. Moreover, the contextual condition of social influence was lacking: *“Unfortunately, we did not get a teacher involved in the work, and there we failed”* (IP 4). Although beliefs about the consequences of implementation were present in two cases, this was insufficient to support the decision to implement the guideline when neither knowledge acquisition nor social influence was present.

### Strategy 3: Ongoing training

Ongoing training led to implementers maintaining their intention to implement the guideline and enacting implementation activities over time. “*It kind of led to … it was not just a project that got started and then was dropped. Because we had these meetings throughout the year, and that made us go, like, ‘Right, we have to do this’”* (IP 14). PDSA documentation also demonstrated the attainment of this outcome, including the specific activities, the individuals responsible, and the planned execution timelines. The documents also revealed that implementers enacted implementation activities between workshops. For this strategy, two distinct pathways were identified: one successful (*n* = 12) and one unsuccessful pathway (*n* = 4).

#### Successful pathway

**Fig. 5:**
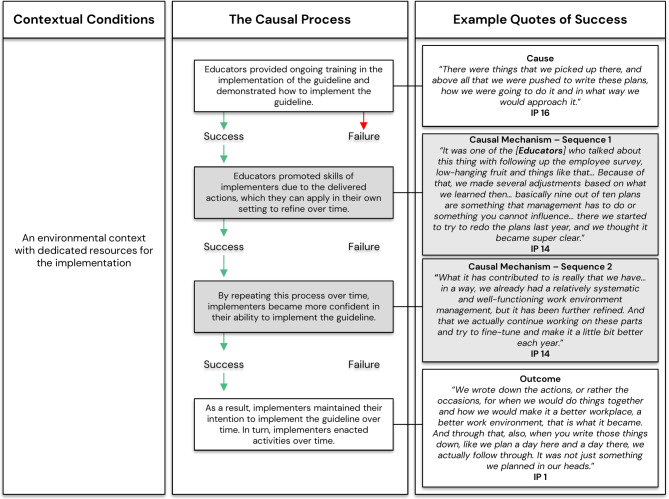
The pathways for the ongoing training. Note: the successful pathway is illustrated with green arrows, while the unsuccessful pathway is illustrated with red arrows. Success = presence; failure = absence. Each part of the pathway is accompanied by an example quote from the data

Figure 5 illustrates the pathway that the implementers went through to achieve the strategy outcome.

For this strategy to function, a context with dedicated resources for implementation was consistently present, reflected in the prioritization of implementation both during workshop sessions and in the school setting: “*We set a clear plan for the year, which we stuck to. And it meant that we met on a few occasions between the workshops*” (IP 14). As shown in Fig. 5, the strategy engaged the mechanism when educators provided ongoing training on implementing the guideline through lectures, exercises, and the introduction of tools for implementation. Following this, the mechanism unfolded through two sequences. First, following the workshop, implementers applied what they had learned in their respective settings, thereby developing their skills. Continued participation in workshops further facilitated the refinement and consolidation of these skills: “*I mean, for us in the beginning, I think we had something—some kind of enormous task—and it really would have been better if we had started with something smaller, something a bit more manageable that you could actually work through properly. Then you would learn the basics—like this planning, how do you actually go about it—and then you would feel like, ‘Oh, right, that is how it works,’ so the next time you did it in that way*” (IP 1). Second, through repeated participation in workshops, implementers formed beliefs in their capability to implement the guideline: *“And you could also feel, from one workshop to the next, that we became more confident about what we were supposed to do and what it was all about”* (IP 1). This pathway illustrates that the strategy functions as a feedback loop, where practice leads to refinement of skills, which strengthens beliefs in the capability to implement the guideline.

#### Unsuccessful pathways

In the unsuccessful pathway, the strategy failed to engage the mechanism. Specifically, implementers did not develop skills or form beliefs about their capability to implement the guideline. Similarly, the contextual condition of a context with dedicated resources was lacking: “*We probably did not meet all the deadlines, and therefore it did not really have the effect that it might have had if we had … I cannot say that it was about us not being engaged, because we* were *engaged. But I think rather that it just gets eaten up by everyday life priorities*” (IP 15).

### Strategy 4: small cyclical tests of change

The outcome traced for this strategy was behavioral regulation (i.e., when the strategy was successful, it led to behavioral regulation, or the processes by which teams regulate the implementation and ensure that implementers know how, when, and where to contribute to it). One implementer noted, “*It is not just about writing a goal, but also about defining the first step, what needs to be done, in what order, who does what, when it should be finished, and how you follow up*” (IP 13). The presence of behavioral regulation was echoed in the PDSA documentation, which provided evidence of how teams planned, monitored, and adjusted their activities over time. Moreover, the documents showed that the implementers engaged in this process repeatedly. For this strategy, three distinct pathways were identified: two successful (*n* = 4 and *n* = 6) and one unsuccessful pathway (*n* = 6).

#### Successful pathway

**Fig. 6:**
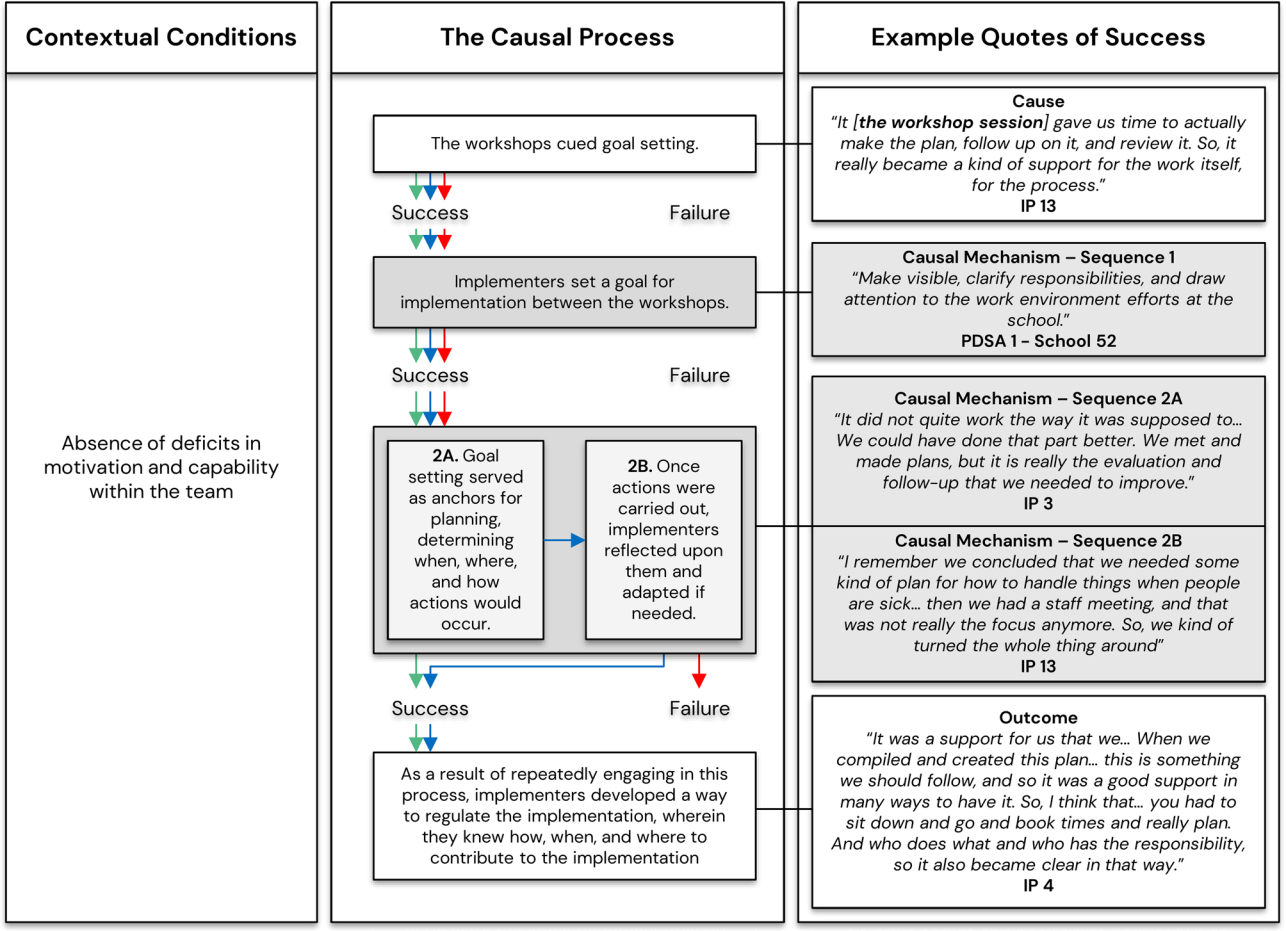
The pathways for the small cyclical tests of change. Note: the successful pathways are illustrated with green and blue arrows, while the unsuccessful pathway is illustrated with red arrows. Success = presence; failure = absence. Each part of the pathway is accompanied by an example quote from the data

Figure 6 illustrates the pathways that the implementers went through to achieve behavioral regulation.

As shown in Fig. 6, these successful pathways were conditional on the absence of two barriers: deficits in motivation and capability within the team. For example, “*we have not had the opportunity to work with it … with this model*” (IP 10), or “*we did not understand, it was a bit unclear, it was kind of like, what is this now, what are we doing now*” (IP 11). The strategy engaged the mechanism when workshops provided time for goal and activity specification. Following this, the mechanism unfolded through two sequences. First, implementers engaged in goal setting by defining and specifying implementation goals between workshops. Second, to achieve the specified goals, implementers conducted action planning, during which teams specified what, when, where, and how activities would be carried out. Once action plans were implemented, teams engaged in reflection, which sometimes led to adaptation in subsequent cycles. As the PDSA cycles were repeated, implementers used this approach to regulate the implementation process, representing one of the successful pathways to the traced outcome. An alternative pathway was also identified in which implementers engaged in goal setting and action planning but did not follow up or reflect on their process: “*It is always this thing with follow-up afterward … that schools and, I am sure, many others [organizations] fail at*” (IP 5). This illustrates that this strategy leads to the implementation being regulated either by the PDSA method, or through repeated goal setting and action planning that left out the ‘study’ and ‘act’ phases of PDSA.

#### Unsuccessful pathways

In the unsuccessful pathway, goal setting and action planning were present. However, the goals and plans remained static, with the PDSA documents showing little or no modification of activities over time. At the same time, barriers such as deficits in motivation and capability within the team were present, which explained why this pathway did not achieve behavioral regulation.

### Strategy 5: implementation facilitation

#### Introduction to the pathways

When implementation facilitation functioned as intended, it supported implementers in goal setting and prioritization. Implementers’ accounts evidenced this outcome: “*We have worked a lot with clarifying the documents [Guideline Recommendation 1] … from the municipality … with the help of [Facilitator]*” (IP 9). The attendance lists supported implementers’ accounts by providing concrete evidence of the facilitator’s participation. One successful (*n* = 7) and two unsuccessful pathways (*n* = 5 and *n* = 4) were identified.

#### Successful pathway

**Fig. 7:**
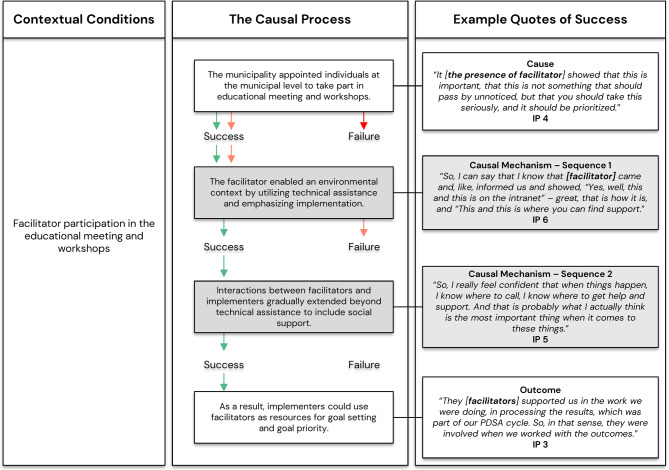
The pathways for facilitation. Note: the successful pathway is illustrated with green arrows, while the two unsuccessful pathways are illustrated with orange and red arrows, respectively. Success = presence; failure = absence. Each part of the pathway is accompanied by an example quote from the data

Figure 7 illustrates the pathways of the facilitation strategy.

This pathway operated on the presence of facilitator participation in the educational meeting and workshops. As shown in Fig. 7, the strategy engaged the mechanism through the municipality’s decision to appoint individuals at the municipal level to take part in these activities. Following this, the mechanism unfolded through two sequences. First, facilitators contributed to creating an environmental context by using their expertise to provide technical assistance. Examples of accounts supporting this sequence include “*It concerned the employee survey, and we were given an overview of how to interpret it, how we should handle the material*” (IP 3). This practical support enabled implementers to leverage municipality structures in their work. Second, interactions between facilitators and implementers gradually extended beyond technical assistance to include social support. This was evident as facilitators became trusted contacts for support and guidance: “*I have a feeling that I know I can turn to [Facilitator] … if it is something that concerns these issues*” (IP 3).

#### Unsuccessful pathways

Two unsuccessful pathways were identified. In the first, the strategy was fully delivered, but failed to engage the mechanism. Although facilitators participated in the educational meeting and workshops, their role was passive, limited to observing the implementers’ work: *“[Facilitator] was there for some of your presentations, absolutely … but after that, she has not been involved. And it is not that the support has not existed when I say nonexistent, but it has not guided or supervised us*” (IP 16). In the second unsuccessful pathway, the strategy was not fully delivered, and therefore, the mechanism could not be engaged. Although the facilitator was present at the educational meeting, both accounts and attendance lists revealed that the appointed facilitator was absent from the workshop series.

## Discussion

This study aimed to explain how a multifaceted implementation strategy for implementing a guideline for the prevention of mental health problems in the workplace operated by identifying the mechanisms engaged by its five discrete strategies. Overall, the findings show that the effectiveness of the multifaceted implementation strategy was dependent on a set of mechanisms engaged by the discrete strategies that unfolded under specific contextual conditions. These mechanisms led to specific proximal outcomes that, combined, contributed to the implementation of the guideline. Specifically, *Organizing Implementation Teams* enabled participation in implementation activities; *Educational Meeting* influenced the decision to implement the guideline; *Ongoing Training* supported the intention to perform and enact implementation activities; *Small Cyclical Tests of Change* influenced behavioral regulation; and *Implementation Facilitation* supported goal setting and prioritization. This study extends previous quantitative work within the trial [19] and strengthens findings by providing qualitative knowledge explaining how and under which contextual conditions guideline fidelity was produced [63].

Our finding that the educational strategies (educational meeting and ongoing training) engaged mechanisms comprising capability and motivation aligns with previous research on educational strategies [50–53]. Moreover, these findings provide a plausible explanation for the quantitative improvements in knowledge, skills, and motivation-related domains of TDF observed in the trial [19]. The educational meeting functioned as a starting point for implementation by prompting a decision to implement the guideline, in line with early implementation outcomes such as adoption [48]. In addition, ongoing training supported continued implementation by enabling implementers to maintain their intention and carry out implementation activities. Together, these strategies lead to an initial decision to implement the guideline, followed by maintained intention and enactment of implementation activities, further contributing to the progression toward guideline fidelity. These mechanisms, however, depend on the presence of contextual conditions that enable these sequences to unfold, indicating that changes in capability and motivation need to be supported by the surrounding context.

Building on this, the mechanisms identified for each strategy unfolded under specific contextual conditions, especially through implementers’ opportunity. The study findings, for example, show that strategies such as organizing implementation teams and implementation facilitation ensured the presence of organizational and social conditions in which other mechanisms, such as those of the educational meeting and ongoing training, could operate. This suggests that the effectiveness of the multifaceted strategy depended not only on the discrete strategies themselves, but on how they were combined to enable and reinforce one another [6, 7]. For example, organizing implementation teams enabled staff involvement, which was necessary for the mechanisms of the educational meeting to unfold. Implementers’ accounts showed that the presence of cross-level collaboration supported the decision to implement the guideline. Consistent with prior systematic reviews on implementation strategies, these findings indicate that while educational strategies may strengthen capability and motivation, but without the presence of organizational conditions, implementers may not achieve intended outcomes [64, 65].

In the trial, the quantitative findings showed that behavioral regulation and goals were among the strongest mediators of fidelity [19]. Our qualitative findings explain this pattern further by showing how cyclical tests of change enabled implementers to regulate their implementation process through goal setting, planning, structuring, and follow-up. Previous research describes that behavioral regulation in this form acts as a bridge between education and enactment of the implementation [66–68]. In the present study, however, several implementers engaged in repeated goal setting and action planning but left out the reflection (Study) and adaptation (Act) phases of the PDSA method. Despite this, they still achieved the outcome of behavioral regulation. This suggests that continuous planning (Plan) and doing (Do) may be necessary to regulate the implementation process, whereas the Study and Act phases may strengthen the quality of implementation without being necessary for implementation to progress [69, 70].

### Contribution to the study of implementation strategy mechanisms

The methods used in this study offer an approach to identifying and assessing the concrete sequence of actions and interactions that explain how and why a strategy works, thereby advancing implementation strategy mechanisms and effectiveness research in three important ways.

First, the granular and sequential knowledge generated through this approach enables more precise specification of how the multifaceted strategy functions when it succeeds, and why it may fail under certain conditions. This level of detail was missing from our previous study, in which the strategy was assessed for its effect on fidelity via the hypothesized mediators (Fig. 1) [19]. Scientifically, this new knowledge can inform the development of measurements and monitoring approaches that are sensitive to whether the strategy unfolds as intended [16, 21]. Practically, this can provide important signals to implementers about potential implementation failure and guide more efficient, context-sensitive deployment of the multifaceted strategy [71, 72].

Second, this study unpacks the hypothesized mechanisms into specific sequences rather than leaving them implicit in assumptions [31, 32]. This, in turn, operationalizes transferable pathways that may be reproduced, depending on whether the underlying mechanisms and contextual conditions can be established. Despite the proliferation of implementation strategy studies, this body of research continues to demonstrate heterogeneous results and small effect sizes [8]. One reason for this is the inconsistent operationalization of the strategies themselves, especially for those that are multifaceted [15, 73, 74]. This study specifies and empirically traces the core strategy actions (e.g., providing education or social restructuring) and the specific sequences required for the mechanism to operate, which produced proximal outcomes that correspond to TDF domains (e.g., behavioral regulation) quantitatively linked to fidelity [19]. The study provides a detailed operationalization of how these proximal outcomes may be reproduced, either through the same or other strategy forms [69, 70, 75]. More broadly, articulating the mechanisms and contextual conditions under which these pathways can be reliably reproduced in other implementation contexts helps build evidence on which aspects of the strategy’s functioning may be generalizable [69].

Third, another persistent challenge in implementation strategy research is the ongoing tension regarding the role of context, specifically what is generalizable versus what is context-specific [69]. Although not entirely unique, structural characteristics of the implementation context, such as principals’ legal responsibility to manage the work environment [76], hierarchical governance structures [77], and the high prevalence of mental health problems among school personnel [78], are context-specific features that may influence how the pathways unfold in this setting and may not be present in other contexts. Our approach allowed us to distinguish between typical and deviant cases to identify the contextual conditions that explain why a case may deviate from a more common path. This allows us to empirically harness contextual complexity and articulate when and what local context matters in explaining why a strategy did not work as intended. These contextual conditions in turn set the boundaries of the transferability of these pathways [63, 79].

### Contribution to school-based implementation research

This study also contributes to the understanding of multifaceted strategies in school settings. First, it specifies which discrete strategies engage which mechanisms, addressing a key gap in school-based implementation research [5]. For example, the identified pathways illustrate how cornerstone strategies such as education and training operate in school settings. Second, it shows how strategies within a multifaceted package can amplify one another’s impact [6, 7]. For example, ongoing training and small cyclical tests of change appeared highly interdependent, echoing meta-analytic evidence that ‘active’ combinations of strategies like training and feedback are effective in schools [55]. While prior research has suggested that this effectiveness stems from such combination, our findings provide insights into why they are effective together. Third, it shows how strategies such as implementation teams and facilitation can actively shape the contextual conditions under which other strategies operate, serving as enablers of implementation activities. In doing so, the study moves beyond the general claim that ‘context matters’ by specifying which aspects of context matter for which strategies, and how one strategy can be intentionally designed to enable the functioning of another.

### Methodological considerations

Despite the small number of implementers, information power was strengthened through purposive and snowball sampling to ensure varied perspectives, theory-guided analysis, and the research team’s contextual familiarity, which facilitated reflective, inductive interviews that allowed for deep inquiry into each participant’s implementation experience while adhering to a consistent structure that aligned with the study’s goals and parameters [59]. The recruitment strategy may, however, have resulted in an over-representation of implementers who were more engaged or positive towards the strategies. Nevertheless, the data reflected variation in experiences, enabling the identification of both successful and unsuccessful pathways. Variation was observed both across and within cases. For example, the same implementer could describe one strategy as functioning well while another did not. This means that successful pathways were not limited to specific implementers.

Furthermore, credibility may have been influenced by potential biases such as social desirability, recall, and variations in work experience [59], particularly given the retrospective nature of the interviews. The findings should therefore be interpreted as implementers’ accounts of these mechanisms, rather than direct observations of cognitive or motivational processes. Credibility was strengthened through prolonged engagement, data triangulation, and peer debriefing [80]. For example, the research team’s involvement with the implementers for almost a year helped build trust and develop a deeper understanding of participants’ perspectives and the implementation context. This familiarity with both the implementers and the context enabled the research team to better contextualize and interpret participants’ accounts. However, these strategies cannot fully mitigate the risk of retrospective reconstruction or socially desirable responses.

Finally, the results should be interpreted in light of the theoretical foundations of this study, namely COM-B and TDF. This foundation guided our interpretation of the mechanisms described in the PTOC. However, like all theories, they represent simplified versions of complex realities and should be seen as evolving rather than final. Future research can confirm, modify, or challenge our interpretations, especially as other contexts or perspectives could suggest different but equally plausible pathways.

## Conclusion

We identified five successful pathways explaining how, why, and under what conditions five discrete implementation strategies contribute to implementing a guideline for the prevention of mental health problems in Swedish schools. This study extends previous findings from the same trial [19] by outlining the mechanisms that explain how and why the multifaceted strategy leads to proximal outcomes that contribute to progression toward the implementation outcome of fidelity. This study provides a detailed account of how each discrete strategy worked, why it worked, and when strategies failed. This study presents a rigorous and replicable methodological approach that illustrates how an intentional selection and integration of qualitative methods can be used in implementation strategy research.

## Electronic supplementary material

Below is the link to the electronic supplementary material.

Supplementary Material 1

## Data Availability

The data collected for the current study are not publicly available.

## Abbreviations

HR: Human Resources
EBP: Evidence-Based Practice
PDSA: Plan-Do-Study-Act
PTOC: Process Theory of Change
TDF: Theoretical Domains Framework
QCA: Qualitative Comparative Analysis
EQCA: Extractive Qualitative Content Analysis
