# Supplementary material for: The roads towards fidelity: a mixed-method study of mechanisms of a multifaceted implementation strategy to improve implementation of a guideline for the prevention of mental Ill-health at the workplace in a school setting
Source: Implement Sci Commun. 2026 Jun 11;7:114. doi: 10.1186/s43058-026-01010-0 (PMC13261927; doi:10.1186/s43058-026-01010-0)
Supplement: Supplementary file 1 — Supplementary Material 1 [file 43058_2026_1010_MOESM1_ESM.docx]

**Appendix 1**

| **Strategy** | **Categories** | **Dimensions** |
| --- | --- | --- |
| **Implementation Teams** | | |
| **Sequence 1** | Environmental Context and Resources | Resources (Facilitator) |
| **Sequence 2** | Social Influence | Social Support |
|  |  | Group Norms **(New)** |
| **Outcome** | Social/professional role and identity (**New**) |  |
| **Contextual conditions** | Environmental Context and Resources | Priorities (Barrier) |
|  |  | Staff sick leave/turnover (Barrier) |
| **Educational Meeting** | | |
| **Sequence 1** | Knowledge |  |
| **Sequence 2** | Beliefs about Consequences |  |
|  | ~~Beliefs about Capability~~ |  |
| **Outcome** | Goals, ~~intention~~ |  |
| **Contextual Conditions** | Social Influence | Social Support |
| **Ongoing Training** | | |
| Sequence 1 | Knowledge |  |
|  | Skills |  |
| Sequence 2 | ~~Beliefs about Consequences~~ |  |
|  | Beliefs about Capability |  |
| Outcome | Intention |  |
|  | Enactment **(New)** |  |
| Context | Environmental Context and Resources | Priorities (Determinant) |
| **Small Cyclical Test of Change** | | |
| Sequence 1 | Goals |  |
| Sequence 2 | Action Planning |  |
|  | Self-Regulation |  |
| Outcome | Behavioral Regulation |  |
| Context | Barriers (Env.Con/Resources) | Capability (Barrier) **(New)** |
|  |  | Motivation (Barrier) **(New)** |
| **Facilitation** | | |
| Sequence 1 | Environmental Context and Resources | Resources (Facilitator) |
| Sequence 2 | Social Influence | Social Support |
| Outcome | Goals |  |
| Context | Env.Con/Resources | Resources (Barrier) |

| **Categories** | |  |
| --- | --- | --- |
| **Knowledge** | |  |
| **Definition** | An awareness of the existence of something (Atkins et al., 2017). |  |
| **Categorize the extracted information as knowledge if:** | |  |
| Information describing changes in awareness of mental health/ill-health, the guideline, or preventive work. For example, general knowledge about the topic or the intervention includes know-how knowledge, i.e., how to follow a routine or carry out a task, as well as understanding the context in which a task is performed. | |  |
| **Example quote:** "Before the educational meeting, I knew there were regulations on how we should handle psychosocial risks. During the day, I also learned how to work preventively." | |  |
| **Indicate the point in time the information refers to:** | |  |
| **Time** | **For example** * Pre-Implementation * Active-Implementation * Unclear |  |
| **Categorize the extracted information as:** | |  |
| **Content** | Information about the content of this category. |  |
| **Reported Causes** | Information about the action that led to knowledge. |  |
| **Reported Effect** | Information about the effect that the knowledge led to. |  |
| **Skills** | |  |
| **Definition** | An ability or proficiency acquired through practice (Atkins et al., 2017). |  |
| **Categorize the extracted information as skills if:** | |  |
| Information describing changes in: Skills; Professional development; Competence; Ability to implement the guideline. | |  |
| **Example quote:** "When we began setting concrete goals for managing stress in the workplace, it became clearer to me what I actually needed to do in practice to implement the guidelines." | |  |
| **Indicate the point in time the information refers to:** | |  |
| **Time** | **For example** * Pre-Implementation * Active-Implementation * Unclear |  |
| **Categorize the extracted information as:** | |  |
| **Content** | Information about the content of this category. |  |
| **Reported Causes** | Information about the action that led to the skills. |  |
| **Reported Effect** | Information about the effect that the skills led to. |  |
| **Intention** | |  |
| **Definition** | A conscious decision to perform a behaviour or a resolve to act in a certain way (Atkins et al., 2017). |  |
| **Categorize the extracted information as intention if:** | |  |
| Information describing a conscious decision to implement the guideline or to work preventively with the work environment. This differs from "goals," as this category captures a decision to act, whereas goals are about concrete actions (such as making a plan). For example, a consistent intention (e.g., stability of intention) to perform a specific behaviour over time. | |  |
| **Example quote:** "After the training, we decided that we really would continue using the new routine to follow up on workplace risks." | |  |
| **Indicate the point in time the information refers to:** | |  |
| **Time** | **For example** * Pre-Implementation * Active-Implementation * Unclear |  |
| **Categorize the extracted information as:** | |  |
| **Content** | Information about the content of this category. |  |
| **Reported Causes** | Information about the action that led to the intention. |  |
| **Reported Effect** | Information about the effect that the intention led to. |  |
| **Goals** | |  |
| **Definition** | Mental representations of outcomes or end states that an individual wants to achieve (Atkins et al., 2017). |  |
| **Categorize the extracted information as goals if:** | |  |
| Information describing conscious actions or representations to implement the guideline, such as respondents stating that they have an action plan or goals for implementation at the school. For example, respondents described: Goals (long-term/short-term); goal setting; prioritizing goals; planning how, when, and where a behavior should be performed; and a concrete plan linking a situation to a behavior. | |  |
| **Example Quote:** "We set a goal to carry out at least two activities focused on psychosocial factors before the end of the term. That gives us something concrete to work toward and follow up on." | |  |
| **Indicate the point in time the information refers to:** | |  |
| **Time** | **For example** * Pre-Implementation * Active-Implementation * Unclear |  |
| **Categorize the extracted information as:** | |  |
| **Content** | Information about the content of this category. |  |
| **Reported Causes** | Information about the action that led to Goals. |  |
| **Reported Effect** | Information about the effect that the Goals led to. |  |
| **Beliefs about Consequences** | |  |
| **Definition** | Acceptance of the truth, reality, or validity about outcomes of a behaviour in a given situation (Atkins et al., 2017). |  |
| **Categorize the extracted information as Beliefs about Consequences if:** | |  |
| Information describing that the respondent gained increased belief/understanding of the importance of implementing the guideline/working with mental health or systematically managing risks. Examples of descriptions of beliefs or convictions about a certain behaviour: What a person believes will happen if they perform a certain behaviour; whether consequences are seen as positive/negative, actual or perceived outcomes of a behaviour. | |  |
| **Example Quote:** "The educational meeting made me really understand why it is so important that we follow the guideline." | |  |
| **Indicate the point in time the information refers to:** | |  |
| **Time** | **For example** * Pre-Implementation * Active-Implementation * Unclear |  |
| **Categorize the extracted information as:** | |  |
| **Content** | Information about the content of this category. |  |
| **Reported Causes** | Information about the action that led to Beliefs about Consequences. |  |
| **Reported Effect** | Information about the effect that Beliefs about Consequences led to. |  |
| **Beliefs about Capability** | |  |
| **Definition** | Acceptance of the truth, reality or validity about an ability, talent or facility that a person can put to constructive use (Atkins et al., 2017). |  |
| **Categorize the extracted information as Beliefs about Capability if:** | |  |
| Information describing that the respondent gained increased belief/understanding in their ability to implement the guideline. For example, information describing: Self-confidence; Perceived competence; Self-efficacy or belief in own ability; Perceived control over behaviour; Empowerment | |  |
| **Example Quote:** At first, I felt unsure whether I could really lead the implementation of the guideline – it felt like too big a responsibility. However, now that we've had support and worked with it step by step, I actually feel that I've the ability to drive it forward. | |  |
| **Indicate the point in time the information refers to:** | |  |
| **Time** | **For example** * Pre-Implementation * Active-Implementation * Unclear |  |
| **Categorize the extracted information as:** | |  |
| **Content** | Information about the content of this category. |  |
| **Reported Causes** | Information about the action that led to beliefs about capability. |  |
| **Reported Effect** | Information about the effects of beliefs about capability. |  |
| **Social Influence** | |  |
| **Definition** | Those interpersonal processes that can cause individuals to change their thoughts, feelings, or behaviours (Atkins et al., 2017). |  |
| **Dimensions** |  |  |
| **Categorize the extracted information into (dimension) if:** | |  |
| **Social Support:** Information describing the respondent's experience of perceived support from colleagues, management, or others | |  |
| **Group Norms:** Information describing the respondent's experience of Norms specific to a workgroup or context | |  |
| **Indicate the point in time the information refers to:** | |  |
| **Time** | **For example** * Pre-Implementation * Active-Implementation * Unclear |  |
| **Categorize the extracted information as:** | |  |
| **Content** | Information about the content of this category/dimension. |  |
| **Reported Causes** | Information about the action that led to the dimension. |  |
| **Reported Effect** | Information about the effects of the dimension. |  |
| **Environmental Context/resources** | |  |
| **Definition** | Any circumstance of a person's situation or environment that discourages or encourages the development of skills and abilities, independence, social competence and adaptive behaviour (Atkins et al., 2017). |  |
| **Dimensions** | |  |
| **Categorize the extracted information into (dimension) if:** | |  |
| **Barriers:** Information describing circumstances that hindered implementation, but the absence of these circumstances does not automatically lead to a positive influence on implementation. | |  |
| **Facilitators:** Information describing circumstances that promoted implementation, but the absence of these circumstances does not automatically lead to a negative influence on implementation. | |  |
| **Determinants:** A factor that can both positively or negatively affect an implementation outcome. | |  |
| **Time** | **For example** * Pre-Implementation * Active-Implementation * Unclear |  |
| **Categorize the extracted information as:** | |  |
| **Content** | Information about the content of this category/dimension. |  |
| **Reported Causes** | Information about the action that led to the dimension. |  |
| **Reported Effect** | Information about the effects of the dimension. |  |
| **Behavioral regulation** | |  |
| **Definition** | Anything aimed at managing or changing objectively observed or measured actions (Atkins et al., 2017). |  |
| **Categorize the extracted information as Behavioral regulation if:** | |  |
| Information describing efforts to self-monitor, plan, adjust, or maintain behaviours related to the implementation of the guideline. This includes: action planning, self-regulation, and/or breaking existing habits. | |  |
| **Example Quote:** "We made a plan at the workshop. It helped keep us focused and not drop it when things got hectic." | |  |
| **Time** | **For example** * Pre-Implementation * Active-Implementation * Unclear |  |
| **Categorize the extracted information as:** | |  |
| **Content** | Information about the content of this category/dimension. |  |
| **Reported Causes** | Information about the action that led to the dimension. |  |
| **Reported Effect** | Information about the effects of the dimension. |  |
| **Social professional role/identity** | | |
| **Definition** | | A coherent set of behaviours and displayed personal qualities of an individual in a social or work setting (Atkins et al., 2017). |
| **Dimensions** | |  |
| **Categorize the extracted information into social professional role/identity if:** | | |
| Information describing the respondent's experience of formal or informal opportunities and commitment to influence decision-making and a sense of belonging with a group by participating in the implementation. | | |
| **Indicate the point in time the information refers to:** | | |
| **Time** | | **For example** * Pre-Implementation * Active-Implementation * Unclear |
| **Categorize the extracted information as:** | | |
| **Content** | | Information about the content of this category/dimension. |
| **Reported Causes** | | Information about the action that led to the dimension. |
| **Reported Effect** | | Information about the effects of the dimension. |
